# Estimation of a New Canopy Structure Parameter for Rice Using Smartphone Photography

**DOI:** 10.3390/s20144011

**Published:** 2020-07-19

**Authors:** Ziyang Yu, Susan L. Ustin, Zhongchen Zhang, Huanjun Liu, Xinle Zhang, Xiangtian Meng, Yang Cui, Haixiang Guan

**Affiliations:** 1 School of Public Administration and Law, Northeast Agricultural University, Harbin 150030, China; ziyangyu@yeah.net (Z.Y.); liuhuanjun@neigae.ac.cn (H.L.); mxt0123neau@yeah.net (X.M.); yangcuic@yeah.net (Y.C.); ghx624327095@163.com (H.G.); 2 Center for Spatial Technologies and Remote Sensing (CSTARS), John Muir Institute of the Environment, University of California, Davis, CA 95616, USA; slustin@ucdavis.edu; 3 College of Agriculture, Northeast Agricultural University, Harbin 150030, China; zzcneau@neau.edu.cn; 4 Northeast Institute of Geography and Agroecology, Chinese Academy of Sciences, Changchun 130102, China

**Keywords:** canopy structure, digital image, image segmentation, random forest, rice

## Abstract

The objective of this study was to develop a low-cost method for rice growth information obtained quickly using digital images taken with smartphone. A new canopy parameter, namely, the canopy volume parameter (CVP), was proposed and developed for rice using the leaf area index (LAI) and plant height (PH). Among these parameters, the CVP was selected as an optimal parameter to characterize rice yields during the growth period. Rice canopy images were acquired with a smartphone. Image feature parameters were extracted, including the canopy cover (CC) and numerous vegetation indices (VIs), before and after image segmentation. A rice CVP prediction model in which the CC and VIs served as independent variables was established using a random forest (RF) regression algorithm. The results revealed the following. The CVP was better than the LAI and PH for predicting the final yield. And a CVP prediction model constructed according to a local modelling method for distinguishing different types of rice varieties was the most accurate (coefficient of determination (R^2^) = 0.92; root mean square error (RMSE) = 0.44). These findings indicate that digital images can be used to track the growth of crops over time and provide technical support for estimating rice yields.

## 1. Introduction

Crop yield is determined predominantly by photosynthesis of the crop canopy. Accordingly, the interception and use of light energy by the canopy are important factors in determining yield [1,2,3]. The leaf area index (LAI) and plant height (PH) are important indices for characterizing crop canopy structure, which affects yield production and the accumulation of photosynthesis products. The LAI, defined as the plant leaf area per unit of ground surface area [4], affects the final yield by influencing the distribution, interception and use of light energy [5,6,7]. In contrast, PH is an important index for characterizing the competitive ability of plants to maintain a beneficial position within the canopy for the absorption and use of light energy [8], and PH is strongly correlated with both biomass and grain yield [9]. Both the LAI and PH represent the competitive ability of crop plants to secure resources for growth. However, these two variables represent only the aboveground unidirectional morphological characteristics of crops. Moreover, the LAI and PH are limited in terms of their accuracy of final yield predictions.

The LAI is measured mainly by the grid calculation method or by a canopy analyser, while PH is measured manually. These methods are cumbersome and require considerable manpower and material resources [10]. Alternatively, multispectral remote sensing technologies can rapidly and nondestructively measure the growth parameters across a wide range of different types of crops [11]. However, such methods are sensitive to the saturation of measurements caused by dense canopies and thus underestimate canopy growth parameters. Near-surface hyperspectral techniques used for estimating canopy structure parameters can reduce measurement saturation by the use of optimized vegetation indices (VIs) [12,13]. In addition, spatial point cloud information and laser intensity information from light detection and ranging (LiDAR) data can be used to characterize crop canopy structure directly and accurately [14,15]. Nevertheless, multispectral remote sensing technologies and LiDAR sensors are expensive to implement, and their parameter extractions are complex; consequently, these techniques are difficult to promote in actual farmland production systems.

With the increasing popularity of smartphones, digital cameras have become widely available and therefore could be used to address the abovementioned problem. At present, digital cameras are the most convenient tools for acquiring measurements in the visible spectrum and are already widely used for monitoring crop growth. Adamsen et al. reported that the G/R value was linearly related to the normalized difference vegetation index (NDVI) in experiments in which digital cameras were used to monitor the ageing of wheat [16]. Rorie et al. used digital cameras to quantify corn leaf greenness values and extracted hue, saturation and brightness values from digital images; the authors further calculated a dark green colour index (DGCI) to establish a linear relationship between the DGCI and yield [17]. Lee and Lee. extracted image feature parameters and various VIs as independent variables and used a stepwise multiple linear regression (SMLR) model to predict the LAI, aboveground dry weight, nitrogen accumulation and other parameters of rice [18]. More recently, Wang et al. used rice canopy cover (CC) information extracted from digital images instead of the aboveground dry weight to determine critical N concentrations [19].

With the gradual emergence of the era of artificial intelligence, some machine learning algorithms have been applied to analyze remote sensing data. For example, the random forest (RF) regression model, which was proposed by the American statistician Breiman, is an integrated learning model based on multiple classification trees [20]. This model is widely used in remote sensing to solve classification and regression problems [21,22].

The LAI and PH are important structure indicators for describing crop growth. Explaining the influences of canopy structure parameters on yield is highly important. Moreover, digital cameras and image processing technologies have numerous advantages, including low costs and convenient operation, for monitoring the structure parameters of rice canopies. Correspondingly, the purposes of this paper are twofold. First, we aimed to combine two indicators, the LAI and PH, to construct a new canopy structure parameter, namely, the canopy volume parameter (CVP); compare the correlations between rice yield and the LAI, PH, and CVP; and explore the ability of the CVP to indicate rice yield. Second, we aimed to extract feature parameters from cell phone digital images and use the RF regression algorithm to construct a precise CVP estimation model.

## 2. Materials and Methods

### 2.1. Experimental Design

**Figure 1:**
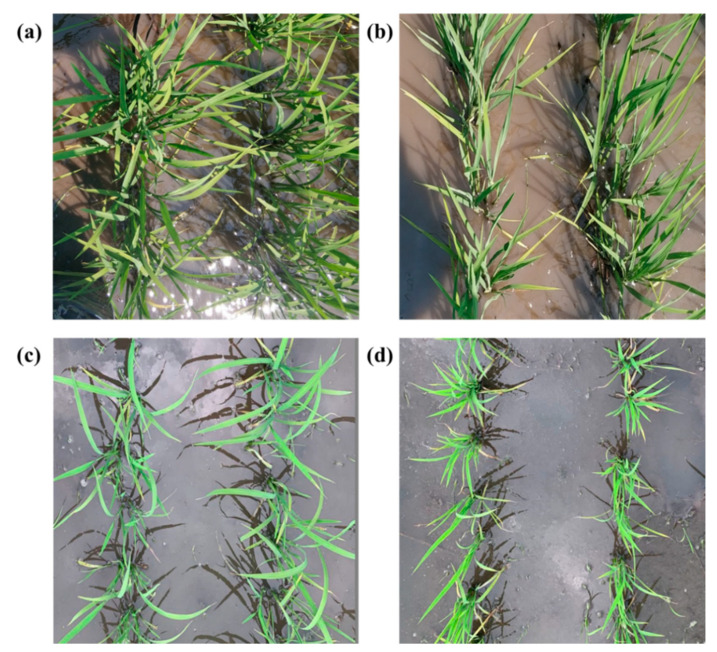
Canopy images of different varieties of rice at the tillering stage: (**a**) SJ18, (**b**) LY16, (**c**) SJ6 and (**d**) WYD4.

Two rice field experiments (Experiments 1 and 2) were conducted at the Fangzheng Test Station (128°13′E, 45°32′N) and the Wuchang Experimental Station (127°12′E, 44°44′N), respectively, in Heilongjiang Province between June and September 2018. The fertilizers used were diammonium, urea and potassium sulfate; the diammonium had a phosphorus content of 46% and a nitrogen content of 17%, the urea had a nitrogen content of 46%, and the potassium sulfate had a potassium content of 50%. Each experiment involved the same five nitrogen rates (0 (N0), 79.1 (N1), 90.5 (N2), 102.3 (N3) and 115 (N4) kg/ha) in accordance with a randomized split-block design with three replications. Two types of rice varieties were used in this study. The first type comprised SJ18 and SJ6, which are early-maturing and high-yield varieties; we denoted these varieties as type A. The second type comprised LY16 and WYD4, which are late-maturing and high-quality varieties that present greater protein and starch contents; we denoted these varieties as type B. The growth period of these two types of rice varieties differs by approximately 7 days. Each experiment included a total of 30 plots, with each plot covering an area of 10 m^2^. The main plot received fertilizer in three split doses: 40% as basal fertilizer applied before transplanting, 30% of fertilizer applied at the tillering stage, and the remaining 30% applied at the stem elongation stage. Details about the experimental design are presented in Table 1. The canopy features of the different rice varieties at the tillering stage are shown in Figure 1. It can be seen that the type A (SJ18 and SJ6) canopy leaves are long and loosely packed together (Figure 1a,c), while the type B (LY16 and WYD4) canopy leaves are short and compact (Figure 1b,d).

### 2.2. Sample Collection and Measurements

A total of 240 samples were obtained at four different growth stages: the tillering stage, jointing stage, heading stage, and filling stage. The sampling areas were randomly chosen in each plot. The same rice plants were selected in the sampling area of each plot for measuring the LAI and PH at each growth stage. The LAI of each plot was measured with a LAI-2200 plant canopy analyzer (LI-COR Inc., Lincoln, NE, USA), and PH was quantified with a ruler, in which the distance from the base of the stem to the top of the tallest leaf of each rice plant was measured. The average PH of six rice plants in the sampling areas was calculated and constituted the PH of each plot. On the same day, images of the rice canopy were collected with a smartphone (Xiao Mi 6, Xiaomi technology co. LTD, Beijing, China). All 240 images were captured under clear-sky conditions. The phone was positioned 1 m above the top of the canopy. The image acquisition mode was set to auto white balance and auto focus, and the images were stored in joint photographic expert group (JPEG) format at a resolution of 1920 × 1080. The images were collected between 12:00 and 13:00. At maturity, the rice plants were harvested from the central region of each plot within an area of 1 m × 1 m to determine the actual yield.

### 2.3. Proposed Canopy Volume Parameter (CVP)

The LAI is defined as the total leaf area per unit of ground surface area [4]. The LAI was calculated as follows:

LAI = TLA/GA(1)

where TLA is total leaf area of the target area of each plot, GA is ground area of the target area of each plot.

The LAI and PH represent the expansion of plants in different directions and affect the interception and use of light energy by the rice canopy. The product of the LAI and PH comprehensively characterizes the growth of plants and their ability to intercept light energy. Therefore, on the basis of the above, a new canopy structure parameter namely CVP was proposed as follows:

CVP = LAI × PH(2)

where CVP is the canopy volume parameter, LAI is the leaf area index of each plot, and PH is the plant height of each plot.

Combining the formula of Equations (1) and (2), the final formula of CVP can be deduced as follows:

CVP = (TLA/GA) × PH = (TLA × PH)/GA(3)

The TLA represents the leaf area distribution in the horizontal direction, and the PH represents the size of plants in the vertical direction. The CVP can be used as another way to describe the spatial volume of rice canopy per unit of ground surface area.

At the heading stage, the rice canopy structure reached its maximum value, and the canopy was considered fully developed. Accordingly, this is the key period that determines the number of rice grains produced. Therefore, the LAI, PH, and CVP values at the heading stage (denoted as LAI_HS_, PH_HS_, and CVP_HS_, respectively) and the average LAI, PH and CVP values of all four stages (denoted as LAI_avg_, PH_avg_, and CVP_avg_, respectively) were selected as growth characteristics. Correlation analysis was performed between the values of the above characteristics and the actual rice yield, and the results were used to verify the applicability of the CVP as a canopy structure parameter.

### 2.4. Image Processing Method

#### 2.4.1. Image Segmentation Method

**Figure 2:**
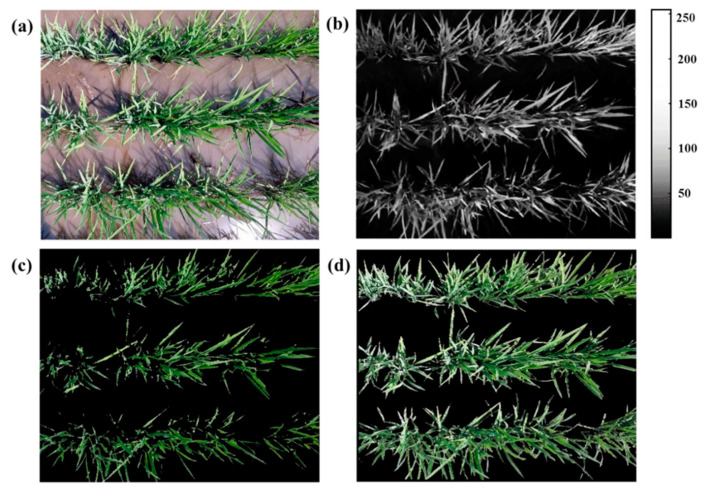
Images of the rice canopy captured by a smartphone and the same images after segmentation: (**a**) original image, (**b**) green channel minus red channel (GMR) value and (**c**,**d**) segmented rice canopy images with GMR thresholds of 30 and 10, respectively.

The RGB pixel values of digital images are different from those of reflectance bands in calibrated satellite-based sensors. The three-band quantization expression represents the relative intensity of each of the reflected red, green, and blue bands [23]. The reflectance of green vegetation in the green band is significantly greater than the reflectance in the red band, while the reflectance values of soil and water in the green and red bands are nearly equal. Therefore, after the digital images were processed by subtracting the red channel reflectance from the green channel reflectance, the difference between canopy areas and non-canopy areas became obvious. In this paper, the green channel reflectance minus the red channel reflectance was defined as the GMR value [24]. Different GMR thresholds were set for the segmentation scheme to distinguish between canopy regions and non-canopy regions. We used MATLAB (The MathWorks, Inc., Natick, MA, USA) to segment the images and extract the image feature parameters. Figure 2b is a scale image plotted on the basis of the GMR values and the gradual change in colour in the image represents the change in GMR value. The GMR threshold for segmentation of the images was set, and pixels with GMR values greater than the threshold were considered part of the rice canopy, with the remaining considered non-canopy regions. When the threshold was 30 (Figure 2c), and along with the non-canopy regions, some canopy regions were also removed. We then set the GMR threshold to 10 to segment the rice canopy image (Figure 2d). After segmentation, the value of CC, which is the ratio of the number of plant pixels to the total number of pixels in a rice canopy image, was calculated. The calculation formula of CC is as follows:

(4) $$\mathrm{CC}=\frac{N_{P}}{N_{sum}}$$

where *N_p_* is the number of plant pixels in the image, and *N_sum_* is the total number of all pixels in the image.

#### 2.4.2. Vegetation Indices Calculations

The intensities in the blue, green and red channels were extracted before and after image segmentation was performed. Regarding the images before and after segmentation, we calculated ten types of VIs for all images. The VIs of the images before segmentation were calculated by the use of all the pixel values in each image, but the VIs of the images after segmentation were calculated by the use of only the pixels of the rice plant in each image. A total of twenty VIs of images before and after segmentation were calculated. The calculation formulas for these ten types of VIs are shown in Table 2.

### 2.5. Model Construction and Evaluation

#### 2.5.1. Different Modelling Methods for the CVP

The correlations between the CVP and all the image feature parameters before and after segmentation were analyzed to determine the independent variables of the model. Two modelling methods were employed: (1) Global prediction modelling (GPM) was used to construct a prediction model for all the rice varieties and evaluate the accuracy of the prediction results, and (2) local prediction modelling (LPM) was used to construct a prediction model for each rice variety and the prediction results of each rice variety were combined to evaluate the prediction accuracy.

#### 2.5.2. Random Forest (RF) Modelling

The image-based CVP prediction model was constructed using a RF model, where the CVP was the dependent variable. We selected ten image feature parameters with CVP correlation coefficients greater than 0.5 before and after segmentation as the independent variables of the RF model. These independent variables included the CC and nine VIs both before and after image segmentation. The RF model is a prediction model composed of multiple decision trees. Two completely random processes were included in the sampling procedure: the first was to bootstrap the samples, and the second was to randomize the independent variables when they were selected. For the training data set, the bootstrap resampling method is used to construct m decision trees by the use of m samples. Each split node in the decision tree is randomly selected from *n* inputs such that the variable space can be completely divided. The average of these decision tree predictions is then taken as the predicted dependent variable [20]. The two random processes described above ensured that the samples for each tree were different during training, thereby preventing overfitting. When training the RF model, we tested different values of the tuning parameters mtry and ntree [33]. The mtry is the number of variables randomly sampled as candidates at each split. The default value of mtry is one third of the total number of predictors; because there were ten independent variables for prediction, the mtry was 3. According to the variable properties, increasing the number of regression trees can increase the stability of the estimated variable results; thus, we set the ntree value to 500. All the data sets of information collected at different growth stages were divided into two data subsets in accordance with a 7:3 ratio between the modelling set (*n* = 168) and the validation set (*n* = 72).

#### 2.5.3. Model Evaluation

The predictive performance of the model was evaluated via the coefficient of determination (R^2^) and the root mean square error (RMSE). The R^2^ and RMSE were calculated as follows:

(5) $$R^{2}=1-\frac{\sum_{i}{\left(y_{i}-y_{i}^{\prime }\right)}^{2}}{\sum_{i}{\left(y_{i}-\bar{y}\right)}^{2}}$$

(6) $$\mathrm{RMSE}=\sqrt{\frac{\sum_{i}(y_{i}-y_{i}^{\prime }{)}^{2}}{n}}$$

where $y_{i}$ and $y_{i}^{\prime }$ are the respective measured and predicted CVP values of sample i, $\bar{y}$ is the arithmetic mean of the CVP and *n* is the number of samples.

## 3. Results

### 3.1. Correlations between Canopy Parameters and Yield

PH_HS_, LAI_HS_ and CVP_HS_ represent the PH, LAI and CVP, respectively, at the heading stage. PH_avg_, LAI_avg_ and CVP_avg_ represent the mean values of the PH, LAI and CVP, respectively, for all four growth stages. Table 3 shows the results of a correlation analysis of the canopy parameters and yield. The three canopy parameters in this study were significantly correlated with yield (*p* < 0.01). At the heading stage, the correlation between CVP_HS_ and yield was the greatest for both rice varieties, and this correlation was stronger than the correlations with PH_HS_ and LAI_HS_. The correlation coefficient of LAI_HS_ with the yield of the A varieties was greater than that of PH_HS_ with the yield of the A varieties, whereas the correlation coefficient of PH_HS_ with the yield of the B varieties was greater than that of LAI_HS_ with the yield of the B varieties. 

**Figure 3:**
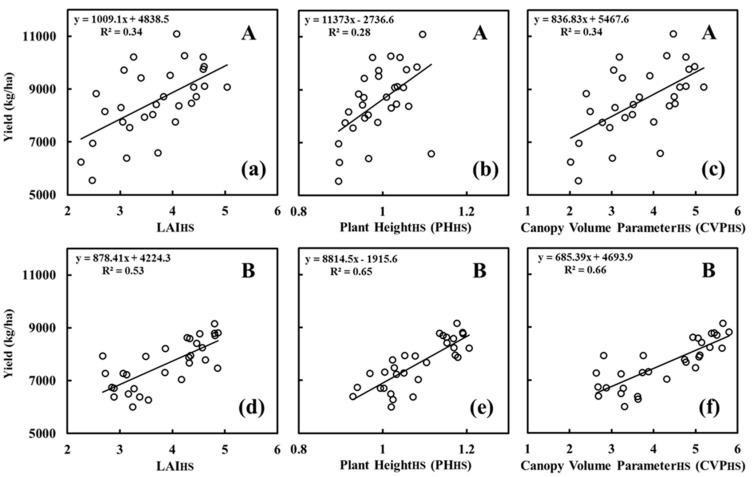

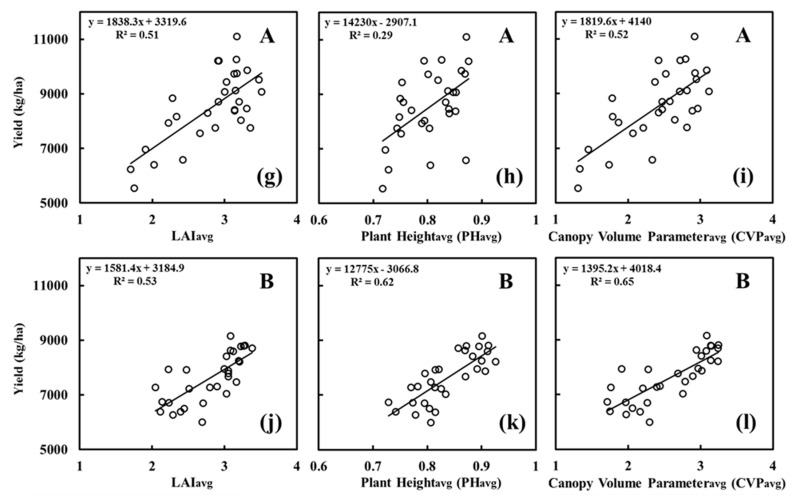
Relationships between canopy parameters and yield. (**a**–**c**) LAI_HS_, PH_HS_ and CVP_HS_ of the A varieties; (**d**–**f**) LAI_HS_, PH_HS_ and CVP_HS_ of the B varieties; (**g**–**i**) LAI_avg_, PH_avg_ and CVP_avg_ of the A varieties; and (**j**–**l**) LAI_avg_, PH_avg_ and CVP_avg_ of the B varieties.

Throughout the entire growth period, the correlations of CVP_avg_ with the yield data from the two types of varieties of rice were stronger than those of PH_avg_ and LAI_avg_. The correlation coefficient between CVP_avg_ and the yield of the A varieties was 0.72, and the correlation coefficient between CVP_avg_ and the yield of the B varieties was 0.81, demonstrating that the CVP is more universally applicable between the two different types of rice varieties. CVP_avg_ was linearly related to yield with R^2^ values that were greater than 0.5 for both types of varieties (Figure 3).

### 3.2. Changes in the CVP at Different Yield Levels during the Whole Growth Period

**Figure 4:**
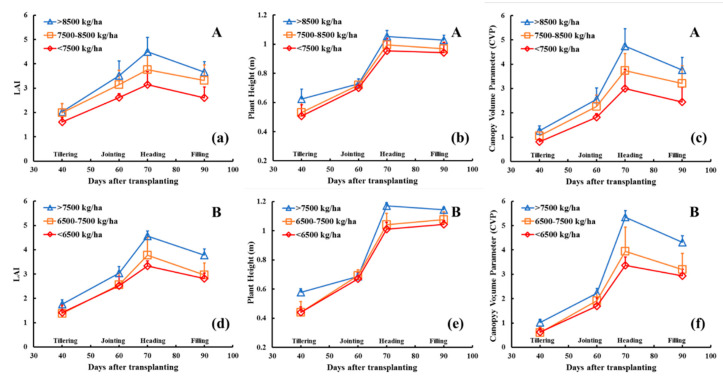
Dynamic changes in rice canopy parameters at different yield levels: (**a**–**c**) LAI, PH and CVP dynamic changes of the A varieties, (**d**–**f**) LAI, PH and CVP dynamic changes of the B varieties.

Different rice varieties have different production capabilities. Hence, we analyzed the yield data of the A varieties and B varieties separately, and the yield was divided into three levels. The dynamic changes in the CVP at different yield levels were then evaluated. Table 4 shows the results of the division of the rice yield data of the different varieties. The dynamic CVP curve (Figure 4) reveals that the trends of CVP values for the different rice varieties were essentially the same. Compared with LAI and PH, the CVP can better indicate yield. 

The values of the high-yield groups of both rice types were greater than those of the middle and low yield groups during the whole growth period. The greater the yield level was, the greater the CVP maximum.

### 3.3. Image Feature Parameters and CVP Correlation Analysis

Table 5 shows the results of the correlation analysis between the image feature parameters and the CVP before and after segmentation. The CC was most strongly correlated with the CVP, and the correlation coefficient was greater than 0.8. Among all the VIs, the INT and NRI were negatively correlated with the CVP before and after segmentation. Before segmentation, all the indices except the NBI, GBRI, and NGBDI presented a correlation coefficient of 0.5 or greater with the CVP. Among these indices, the NGRDI was most strongly correlated with the CVP; the correlation coefficient was 0.7. After segmentation, there were no significant correlations between the CVP and the GBRI, NGBDI, and EXG; however, the other indices were significantly correlated with the CVP (*p* < 0.01). Furthermore, on the basis of a comparison of the correlation coefficients between the VIs and the CVP before and after segmentation, the correlation coefficients between all the VIs (except the NBI) and the CVP before segmentation were greater than those after segmentation.

**Figure 5:**
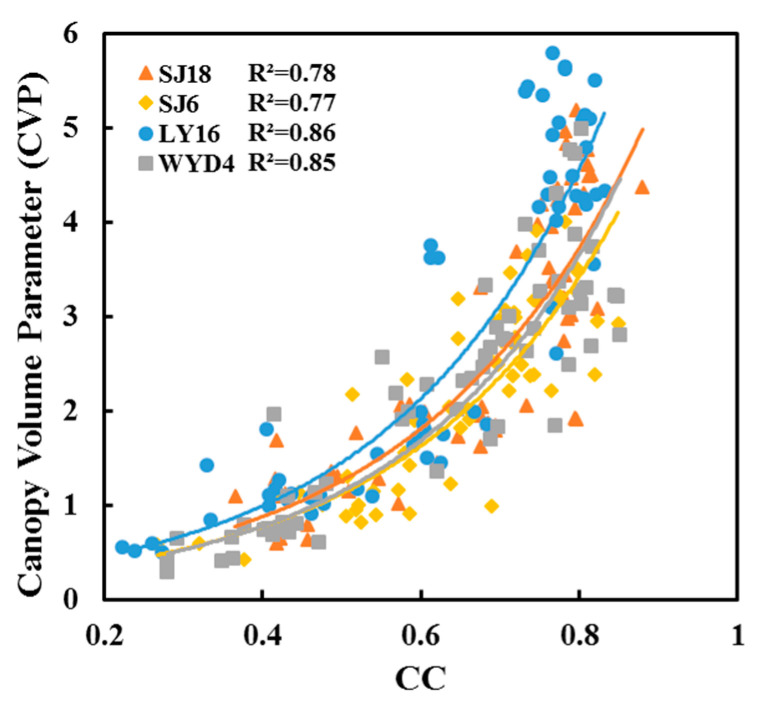
Relationships between the canopy cover (CC) and canopy volume parameter (CVP).

The greatest correlation coefficients among all the image feature parameters occurred between the CC and CVP. Hence, we focused on the analysis of the relationships between the CC and CVP. A scatter plot was constructed (Figure 5) showing that when the CC reached 0.8, the relationship between the CC and CVP became saturated: the CC no longer increased with an increase in the CVP. Owing to the saturation of the CC, the VIs should be considered when constructing a CVP estimation model. Regression analysis further demonstrated that the CC is exponentially related to the CVP (Figure 5), and the following exponential function that best fit the nonlinear relationship was identified:

(7) $$y=ae^{bx}$$

where *y* is the dependent variable representing the CVP, *x* is the independent variable representing the CC, and *a* and *b* represent the parameters obtained by the least-squares method.

The analysis results show that the accuracy of the regression varied between the two different types of rice varieties. The regression accuracy for the B varieties was greater than that for the A varieties. The R^2^ values of the A varieties reached 0.78 and 0.77, and those of the B varieties reached 0.86 and 0.85 (Table 6). Similarly, the trend of the fitting curves for the different rice varieties markedly differed. Therefore, it is necessary to consider the differences in the variety characteristics throughout the growth period to establish a final CVP prediction model.

### 3.4. CVP Prediction Model Based on the RF Model

**Figure 6:**
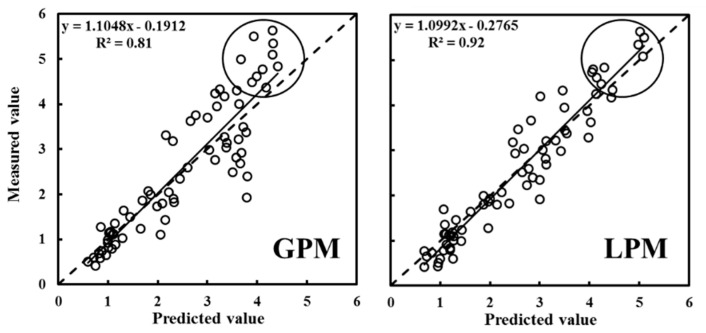
Random forest (RF) regression results for the predicted and measured rice canopy volume parameter (CVP) values (samples for validation): The circled parts in the figures indicate the parts where the CVP measured values become larger.

The independent variables of the RF model included the INT, NGI, NRI, GRRI, NGRDI, EXG, and VDVI before image segmentation and the CC, INT and NRI after image segmentation. The modelling and validation results are shown in Table 7 and Figure 6. On the basis of the results of the verification set, the R^2^ value and RMSE of the global prediction model were 0.81 and 0.66, respectively. Compared with the R^2^ value of the GPM method, those of the LPM method improved by 13.6%, and the RMSE decreased by 33.3%, demonstrating that the LPM accuracy was substantially better. With respect to the LPM method, both the modelling set and validation set R^2^ values were 0.92, and the RMSE differed by only 0.02. According to the scatter plot results for the GPM and LPM methods, when the measured CVP value was greater than 4, for global prediction model, the measured CVP value was significantly greater than the predicted value, whereas for the local prediction model, the predicted CVP value was close to the measured value. These results indicate that the GPM method has poor prediction ability when the actual CVP is large. In contrast, the LPM method solved the saturation problem of the independent variables when the CVP value was greater than 4, and the LPM method was highly accurate and stable.

**Figure 7:**
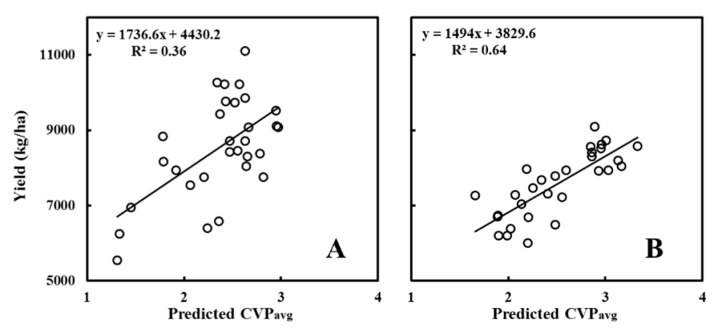
Relationships between the random forest (RF)-predicted CVP_avg_ and yield of the (**A**,**B**) varieties.

The applicability of this method was evaluated by assessing the correlation between the RF-predicted CVP and the yield, as shown in Table 8 and Figure 7. The correlation coefficient between the RF-predicted CVP_avg_ and the yield of the A varieties was 0.6, and the correlation coefficient between the RF-predicted CVP_avg_ and the yield of the B varieties was 0.8.

## 4. Discussion

### 4.1. Advantages of the Use of Digital Imaging for Monitoring Rice Growth

Rice canopy structure parameters can be used to predict final yields. Currently, smartphones are widely used, so the acquisition of digital imagery is easy and inexpensive, but the quality of images obtained by smartphones compared with digital cameras is low. However, our results show that the digital images obtained by smartphones can be used to characterize the structure of rice canopies directly, which is convenient for practical applications in farmland production systems.

### 4.2. Advantages of the Use of CVP Compared with the LAI and PH for Predicting Rice Yields

It has been proven that crop volume, which can be calculated via a 3D model of the crop, is an accurate parameter for predicting yields [34]. The 3D model of a crop can directly provide 3D point cloud data by use of sensors (e.g., LiDAR) [35], or it can be reconstructed from plant images [36]. However, these methods are limited by cost and the working environment, which limits their application under actual field conditions [37]. According to the final formula of CVP in Equation (3), CVP is another way for describing the spatial volume of rice canopy. Compared with the 3D volume, the CVP proposed in this study is easy to obtain, but it is very rough in terms of volume representation. We will gradually improve the CVP in subsequent research. The results also show that the correlation coefficient between CVP and yield of the A varieties was similar to that of LAI, and that between CVP and yield of the B varieties was similar to that of PH. Compared with the correlations of the LAI and PH, the correlation between the CVP and yield was stronger among the different types of rice varieties. The reasons for the above results are as follows. The plant morphology of the A and B rice varieties is different. The canopy leaves of the A rice varieties were relatively long, and the leaves were loosely distributed. On the other hand, the canopy leaves of the B rice varieties were short, and the distribution of leaves was compact (Figure 1). The canopy structures of the different types of rice varieties are heterogeneous [38]. The use of only a single indicator, i.e., either the LAI or PH, to predict rice yields has certain limitations. Rice yields are affected mainly by the photosynthesis production capability from the heading stage to the maturity stage, and this capability depends predominantly on the interception of light energy. LAI can affect the light energy distribution in the horizontal direction of rice canopies. Relatively large LAI values are conducive to the production of rice grains before the heading stage and to the improvement of light energy interception after the heading stage [39,40]. However, the extinction coefficient calculated from solar radiation varies greatly at different heights of the crop canopy, and the extinction coefficient of the top leaf layer is greater than that of the middle and bottom layers [41]. It is necessary to consider the distribution of light energy in the vertical direction to clarify the effects of rice canopy structure on yield. PH is an important morphological trait of crop growth and is strongly related to canopy vertical light distribution [42,43]. Therefore, the CVP can be used to characterize the horizontal and vertical nutrient transport capability and light energy distribution of the rice canopy, and it can reduce the effects of canopy heterogeneity among different rice varieties for predicting yield.

### 4.3. Relationships between Image Feature Parameters and the CVP

Before segmentation, among the various indices, the GRRI and NGRDI were the most strongly correlated with the CVP, and the correlation coefficients were at least 0.65. This is because green vegetation reflects more light in the green band and because more light is absorbed in the red band. Both of the above indices are the ratios of normalized values calculated by the digital numbers (DNs) of the red and green channels; accordingly, these indices can enhance the differences between the reflection characteristics in the green channel and the absorption characteristics in the red channel. The correlations between the VIs before segmentation and the CVP were significantly stronger than those after segmentation, the results of which are similar to those of previous research [24]. The reason for these findings is that before segmentation, the complete image includes the rice canopy and the background; the canopy part of rice is green, and the VIs are calculated by including the average of all pixel DNs. Therefore, as the rice canopy structure becomes larger, the proportion of green pixels in the entire image increases, resulting in increased DNs in the green channel across the entire image. After segmentation, only the canopy part of rice is retained in the image, and all the pixels in the image are green. As the rice canopy structure becomes larger, the total number of pixels in the entire image increases, but the proportion of green pixels in the entire image does not change significantly. Therefore, the VIs before segmentation can effectively characterize the differences in the rice canopy structure. However, in a study estimating the pigments contents in rice canopy leaves, the prediction accuracy of VIs after image segmentation was better than that before segmentation [44]. The images after segmentation include only the canopy portion of rice and thus can be used to directly characterize pigment differences in the rice canopy.

The rice canopy coverage increased when the LAI increased. The CC value extracted from the digital images can directly reflect the value of the LAI from the top view. Among image feature parameters, the CC value was strongly correlated with the LAI [24]. The CVP was constructed by LAI. Therefore, CC is the most important parameter for estimating CVP. Previous studies have shown that CC is exponentially related to LAI [23]. This study showed that the CC also had an exponential relationship with the CVP, and the CC became saturated at 0.8. When the actual CC of rice was greater than 0.8, the extracted CC was slightly less than the actual cover because the leaves at the bottom of the canopy were in the shadows of the top leaves within the canopy; consequently, the leaves at the bottom of the canopy appeared darker in color in the images. 

**Figure 8:**
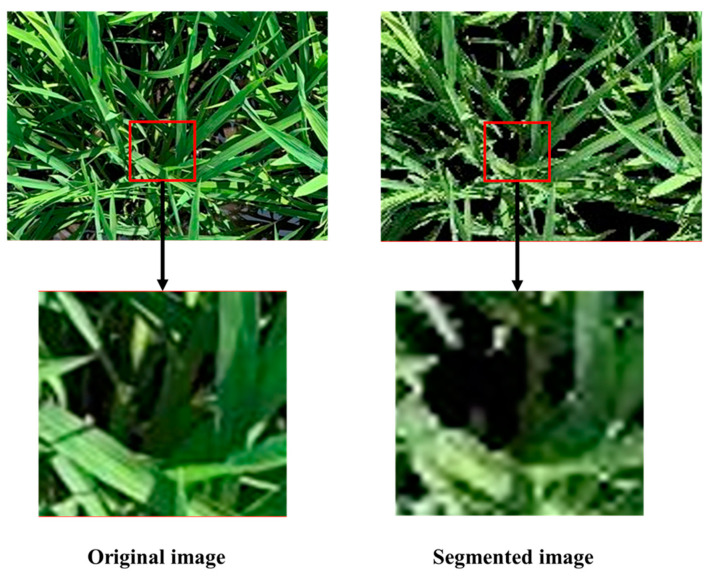
Bottom leaves of the rice canopy below the threshold.

The GMR values of the leaves at the bottom of the canopy fell below the threshold (Figure 8), therefore, these leaves were recognized as the background outside the canopy in the images. When the actual canopy structure increased to a certain degree, the CC no longer increased as the canopy structure became larger. In addition, owing to the heterogeneity of canopy structure among rice varieties, CC could not describe the leaf area distribution and height information of different rice varieties. Therefore, the CC values extracted from the digital images were saturated when predicting the CVP. It is necessary to combine the CC and multiple image features together to estimate the CVP. We used the CC and nine optimal VIs as inputs for our RF model to increase the accuracy. All nine of the optimal VIs are calculated by the DNs of the green and red channels. As the rice canopy structure becomes larger, the DN of the green channel increases, and the DN of the red channel decreases. Therefore, the VIs calculated on basis of the green and red channels can also accurately reflect the size of the rice canopy.

### 4.4. Advantages of the Modelling Methods

Images taken from above the rice canopy and parallel to the ground do not indicate the differences in leaf angles between the different types of varieties. Therefore, the extracted image feature parameters and the CVP response characteristics varied. Similarly, the trends of the regression relationships between the different types of rice varieties for the CC and CVP differed. Global modelling reduced the accuracy of the predictive models without considering the characteristics of the types of varieties. Li et al. reported that the SMLR method is more accurate than the univariate model is at estimating canopy parameters [23]; however, SMLR (a linear regression method) cannot resolve the nonlinear relationships between the image feature parameters and the CVP. According to Liang et al., the use of RF regression to estimate crop canopy indicators was more accurate than was the use of linear fitting and artificial neural networks [22]. As a machine learning algorithm, RF regression boasts significant advantages with respect to the nonlinear fitting of multiple variables and robustly withstands noise. Therefore, the RF algorithm is advantageous because of its high learning efficiency, simple structure, and stability. Moreover, the two randomness strategies of the RF algorithm can solve the problem of overfitting caused by multiple independent variables. Altogether, RF has important advantages for estimating the canopy structure parameters of rice via digital images. Non-destructive rice yield estimation methods include: (1) statistical analysis of the spectral index of remote sensing data and yield [45] and (2) image-based analysis of rice panicle features [46]. However, these methods have some uncertainties. For example, rice spectral data must be obtained under clear and cloudless weather conditions [47]. Moreover, these methods are applied after the emergence of rice panicles, and it is difficult to obtain highly accurate yield estimations before the heading stage. We used the RF-predicted CVP_avg_ to estimate the rice yield reliably. The R^2^ value of the yield prediction accuracy of A varieties is 0.36, and that of B varieties is 0.64. The yield prediction models of all varieties were significant at the 0.01 level of probability (*p* < 0.01). The advantages of our yield estimation method are as follows: (1) the sensor is easy to obtain, and its operation is little affected by weather conditions; (2) the method can diagnose rice growth before the heading stage and guide fertilization in time to obtain target yields.

## 5. Conclusions

This paper involved the use of different varieties of rice in Northeast China as experimental subjects. A new parameter, namely, the CVP, was proposed by combining the LAI and PH. A CVP prediction model was then constructed on the basis of rice canopy feature parameters extracted from digital images, in combination with the RF algorithm. The conclusions are as follows:

(1) The CVP was positively correlated with yield, and the correlation of the CVP with yield was stronger than the correlations of the LAI and PH with yield. Hence, the CVP can be used as an important canopy structure parameter for predicting final yields better than LAI and PH during the rice growth period.
(2) The GMR threshold segmentation method can be used to rapidly segment vegetation and non-vegetation pixels. The correlation between the CC and CVP was the greatest among all the image feature parameters, but when the CVP was large, the CC became saturated. Furthermore, the correlations between the VIs (except the NBI) and CVP before image segmentation were stronger than those after segmentation.
(3) Considering the characteristics of the different types of rice varieties, in combination with the RF regression algorithm, the CVP can be estimated with a high degree of accuracy (R^2^ = 0.92).

## Figures and Tables

**Table 1:** Summary of the experimental design used in this study.

Experiment	Site	Types	Varieties	Rice Variety Characteristics	Nitrogen Rates (kg/ha)
Exp. 1	Fangzheng	A	Suijing18 (SJ18) Longyang16 (LY16)	Early-maturing and high-yield	0 (N0), 79.1 (N1), 90.5 (N2), 102.3 (N3), 115.0 (N4)
		B		Late-maturing and high-quality	
Exp. 2	Wuchang	A	Songjing6 (SJ6) Wuyoudao4 (WYD4)	Early-maturing and high-yield	
		B		Late-maturing and high-quality	

**Table 2:** Vegetation indices (VIs) of images and their calculation formulas.

Index	Formula	Reference
Colour intensity index (INT)	(R + G + B)/3	[25]
Normalized blueness intensity (NBI)	B/(R + G + B)	[26]
Normalized greenness intensity (NGI)	G/(R + G + B)	[26]
Normalized redness intensity (NRI)	R/(R + G + B)	[26]
Green-red ratio index (GRRI)	G/R	[27]
Green-blue ratio index (GBRI)	G/B	[28]
Normalized green-red difference index (NGRDI)	(G − R)/(G + R)	[29]
Normalized green-blue difference index (NGBDI)	(G − B)/(G + B)	[30]
Excess green index (EXG)	2G − R − B	[31]
Visible band difference vegetation index (VDVI)	(2G − R − B)/(2G + R + B)	[32]

**Table 3:** Correlation analysis results of canopy parameters and yield.

Period	Canopy Parameters	A (*n* = 30)	B (*n* = 30)
Heading stage	LAI_HS_	0.58 **	0.75 **
	PH_HS_	0.52 **	0.81 **
	CVP_HS_	0.59 **	0.82 **
Whole growth period	LAI_avg_	0.72 **	0.73 **
	PH_avg_	0.54 **	0.79 **
	CVP_avg_	0.72 **	0.81 **

Note: ** *p* < 0.01. The A rice varieties include SJ6 and SJ18, and the B rice varieties include WYD4 and LY16. The number of samples (*n*) for both types of varieties (A and B) is 30.

**Table 4:** Results of the yield division of the different rice varieties.

Rice Varieties		N0	N1	N2	N3	N4
A	SJ18	Low yield	Middle yield	High yield	High yield	High yield
	SJ6	Low yield	High yield	Middle yield	Middle yield	High yield
B	LY16	Low yield	High yield	High yield	Middle yield	High yield
	WYD4	Low yield	Middle yield	Middle yield	High yield	High yield

Note: The yield of the low-yield group is less than 7500 kg/ha, the yield of the middle-yield group is between 7500 and 8500 kg/ha, and the yield of the high-yield group is more than 8500 kg/ha for the A rice varieties. The yield of the low-yield group is less than 6500 kg/ha, the yield of the middle-yield group is between 6500 and 7500 kg/ha, and the yield of the high-yield group is more than 7500 kg/ha for the B rice varieties.

**Table 5:** Correlation analysis results of image feature parameters and the canopy volume parameter (CVP).

	CC	INT	NBI	NGI	NRI	GRRI	GBRI	NGRDI	NGBDI	EXG	VDVI
Before segmentation		−0.59 **	0.19 **	0.61 **	−0.65 **	0.65 **	0.28 **	0.70 **	0.25 **	0.50 **	0.61 **
After segmentation	0.83 **	−0.54 **	0.26 **	0.22 **	−0.50 **	0.39 **	−0.05	0.46 **	−0.07	−0.02	0.22 **

Note: **, *p* < 0.01.

**Table 6:** Regression analysis results between the canopy volume parameter (CVP) and canopy cover (CC) according to Equation (4).

Type	Varieties	Equation	R^2^
A	SJ18	y = 0.2064e^3.6204x^	0.78
	SJ6	y = 0.1759e^3.7112x^	0.77
B	LY16	y = 0.2158e^3.8165x^	0.86
	WYD4	y = 0.1653e^3.8709x^	0.85

**Table 7:** Canopy volume parameter (CVP) prediction results according to the random forest (RF) methods.

Modelling Method	Modelling Set		Validation Set	
	R^2^	RMSE	R^2^	RMSE
GPM	0.89	0.49	0.81	0.66
LPM	0.92	0.42	0.92	0.44

**Table 8:** Correlations between the RF-predicted CVP_avg_ and yield.

Canopy Parameters	A Varieties	B Varieties
Predicted CVP_avg_	0.60 **	0.80 **

Note: **, *p* < 0.01.
